# Could Mesenchymal Stem Cell-Derived Exosomes Be a Therapeutic Option for Critically Ill COVID-19 Patients?

**DOI:** 10.3390/jcm9092762

**Published:** 2020-08-26

**Authors:** Chiara Gardin, Letizia Ferroni, Juan Carlos Chachques, Barbara Zavan

**Affiliations:** 1 Maria Cecilia Hospital, GVM Care & Research, 48033 Cotignola (RA), Italy; chiara.gardin@unife.it (C.G.); letizia.ferroni@unife.it (L.F.); 2 Department of Morphology, Experimental Medicine and Surgery, University of Ferrara, via Fossato di Mortara 70, 44121 Ferrara, Italy; 3 Department of Cardiac Surgery Pompidou Hospital, Laboratory of Biosurgical Research, Carpentier Foundation, University Paris Descartes, 75015 Paris, France; j.chachques-ext@aphp.fr

**Keywords:** coronavirus, COVID-19, angiotensin-converting enzyme 2, mesenchymal stem cell-derived exosomes, acute lung injury, acute myocardial injury, immunomodulation, anti-inflammation

## Abstract

Coronavirus disease 2019 (COVID-19) is a pandemic viral disease originated in Wuhan, China, in December 2019, caused by the severe acute respiratory syndrome coronavirus 2 (SARS-CoV-2). The severe form of the disease is often associated with acute respiratory distress syndrome (ARDS), and most critically ill patients require mechanical ventilation and support in intensive care units. A significant portion of COVID-19 patients also develop complications of the cardiovascular system, primarily acute myocardial injury, arrhythmia, or heart failure. To date, no specific antiviral therapy is available for patients with SARS-CoV-2 infection. Exosomes derived from mesenchymal stem cells (MSCs) are being explored for the management of a number of diseases that currently have limited or no therapeutic options, thanks to their anti-inflammatory, immunomodulatory, and pro-angiogenic properties. Here, we briefly introduce the pathogenesis of SARS-CoV-2 and its implications in the heart and lungs. Next, we describe some of the most significant clinical evidence of the successful use of MSC-derived exosomes in animal models of lung and heart injuries, which might strengthen our hypothesis in terms of their utility for also treating critically ill COVID-19 patients.

## 1. Introduction

The novel coronavirus 2019 (2019-nCoV) has reached pandemic proportions across the world after originating in Wuhan, the capital of China’s Hubei province, in December 2019 [1,2]. Initially called 2019-nCoV, the World Health Organization (WHO) subsequently adopted the official name severe acute respiratory syndrome coronavirus 2 (SARS-CoV-2) for indicating the virus, and the term coronavirus disease 2019 (COVID-19) for identifying the virus-associated disease [3]. As of 7 May 2020, the WHO has reported almost 3,634,172 confirmed cases of COVID-19 with 251,446 confirmed deaths in 215 countries/areas/territories worldwide [4].

The clinical spectrum of COVID-19 is highly variable—in addition to mild, severe, and critical forms, asymptomatic or paucisymptomatic infections have been described as well [5,6]. Milder clinical conditions are commonly characterized by fever, dry cough, myalgia or fatigue, headache, and mild pneumonia, whereas the severe form of the disease is associated with dyspnea, acute respiratory distress syndrome (ARDS), and hypoxemia (low level of oxygen in arterial blood) [1,7]. The most critical cases experience respiratory failure requiring mechanical ventilation and support in the intensive care units (ICUs) [2,8]. In these patients, systemic manifestations such as septic shock, multiple organ dysfunction (MOD), or multiple organ failure (MOF) often occur as a consequence of the inflammatory cytokine storm triggered by the virus [9]. Huang and colleagues first demonstrated that ICU patients have higher plasma levels of cytokines (e.g., interleukin (IL)-2, IL-6, IL-7, tumor necrosis factor-alpha (TNF-α), granulocyte colony-stimulating factor) and chemokines (e.g., interferon γ-induced protein 10, monocyte chemoattractant protein-1, macrophage inflammatory protein (MIP)-1A) when compared to non-ICU patients [7]. These findings have been confirmed by further studies [10,11].

Similarly to other respiratory tract viral infections, the cardiovascular system may also become involved in several ways, with complications spanning from acute myocardial injury to arrhythmias and exacerbation of heart failure [12,13]. In the study of Wang and coworkers, conducted on 138 patients hospitalized at Zhongnan Hospital of Wuhan University, 16.7% developed arrhythmias and 7.2% developed acute myocardial injury [14]. One possible explanation is the higher cardio-metabolic demand typically correlated to viral infections, which can impair the cardiac function [15]. The other side of the coin is that patients with underlying chronic comorbidities, mainly cardiovascular diseases (CVD) and diabetes, are more likely to be infected. According to currently available epidemiological studies, about 15–30% of COVID-19 patients suffered from hypertension and 2–15% had a history of coronary heart disease [12,16,17]. The severity of primary respiratory syndrome also appears to be accentuated in patients with pre-existing CVD [18].

Although great efforts are being made by scientists and clinicians around the world, no specific antiviral drugs or vaccines have been developed to date. At present, available therapies include non-specific antivirals, antibiotics to treat secondary bacterial infections and sepsis, and systemic corticosteroids to reduce inflammatory-induced lung injury [19]. Overall, these therapies aim to inhibiting the entry of the virus into host cells, replication of its genetic material, and modulation of immune response and inflammation. These treatments have proven to be valuable options for milder forms of the disease; however, they fail in severe cases where the hallmark is the cytokine storm induced by COVID-19 in the lung. In the case of anti-inflammatory medications, their immunomodulatory capacity may not be strong enough when only one or two agents are used, since they can only inhibit specific inflammatory factors [9]. At the same time, some others anti-inflammatory drugs, such as the janus kinase inhibitors (JAK) inhibitors, can also block interferon-alpha (IFN-α) production, which has a key role in fighting the virus. On the other hand, therapeutic use of corticosteroids should be carefully considered, as these further delay the clearance of the virus and even augment the possibility of adverse cardiovascular events [20,21,22]. Other therapies that are under investigation include the employment of antibodies from people who have recovered from COVID-19, the so-called COVID-19 convalescent plasma [23]. Given by transfusion to a patient who is suffering from COVID-19 and with compatible blood type, the donor antibodies bind to SARS-CoV-2, neutralizing the viral particles and blocking their access to uninfected cells, thus possibly shortening the length or reducing the severity of the disease. Though the convalescent plasma treatment has been used for many years, its clinical effectiveness has not been studied extensively. Therefore, this therapeutic option is considered experimental; in fact, the United States Food and Drug Administration (FDA) regulates it as an investigational product, allowing its use for severe or immediately life-threatening COVID-19 infections [24].

Therefore, a safe and effective treatment for COVID-19 patients, particularly for critical cases, is urgently required. The shared idea is that, in addition to inhibiting viral replication, preventing and mitigating the cytokine storm can be the key for saving patients with critical forms of COVID-19. In this regard, human mesenchymal stem cells (MSCs) have been proposed as a suitable therapeutic approach for the management of critical COVID-19 infections. The interest in the use of MSCs for treating COVID-19 patients is undoubtedly huge if we consider that, at the time we are writing, 30 clinical trials have been registered in the national institutes of health (NIH)’s clinical trial database, and 27 clinical trials have begun in China since the onset of the COVID-19 outbreak [25,26].

In this review, we first briefly describe the pathogenesis of SARS-CoV-2 and the implications of COVID-19 in the heart and lungs, then we discuss the potential efficacy of MSC-based immunomodulatory therapy and their secretome in curbing the virus infection. The final part of this review focuses on the clinical evidence of the use of MSC-derived exosomes in the treatment of lung and heart injuries, which might strengthen our hypothesis of their utility also in severe SARS-CoV-2 infections.

## 2. SARS-CoV-2, the Angiotensin-Converting Enzyme 2 Receptor and the Renin-Angiotensin System

SARS-CoV-2 is an enveloped, positive single-stranded RNA virus belonging to the B lineage of the betacoronaviruses. It presents 79.6% sequence identity to the severe acute respiratory syndrome coronavirus (SARS-CoV) implicated in the 2003 SARS outbreak [1]. Like SARS-CoV, the spike glycoprotein (S protein) on the SARS-CoV-2 surface recognizes the angiotensin-converting enzyme 2 (ACE2) receptor for entering into host cells. Zhou and coworkers also proved that SARS-CoV-2 does not use the other coronavirus receptors aminopeptidase N (APN) and dipeptidyl peptidase 4 (DPP4) [1].

As is the case with SARS-CoV, higher ACE2 expression might also correlate with a higher risk of SARS-CoV-2 infection [27]. The ACE2 receptor is widely distributed on the surface of lung alveolar type II (AT2) cells, but it is also abundantly expressed by cells of the heart, blood vessels, liver, digestive organs, and kidneys [28]. In particular, in the heart, ACE2 is localized to cardiomyocytes, cardiac fibroblasts, epicardial adipose tissue, and the coronary vascular endothelium [29]. Such an expression pattern explains why COVID-19 patients, beyond the respiratory syndrome, may also develop cardiovascular damage, acute kidney injury, shock, and death from MOD and MOF syndromes. A recent work by Xu and colleagues based on single cell RNA-sequencing revealed high levels of ACE2 expression also in the epithelial cells of the tongue, suggesting that the oral infection route of SARS-CoV-2 cannot be excluded [30].

**Figure 1:**
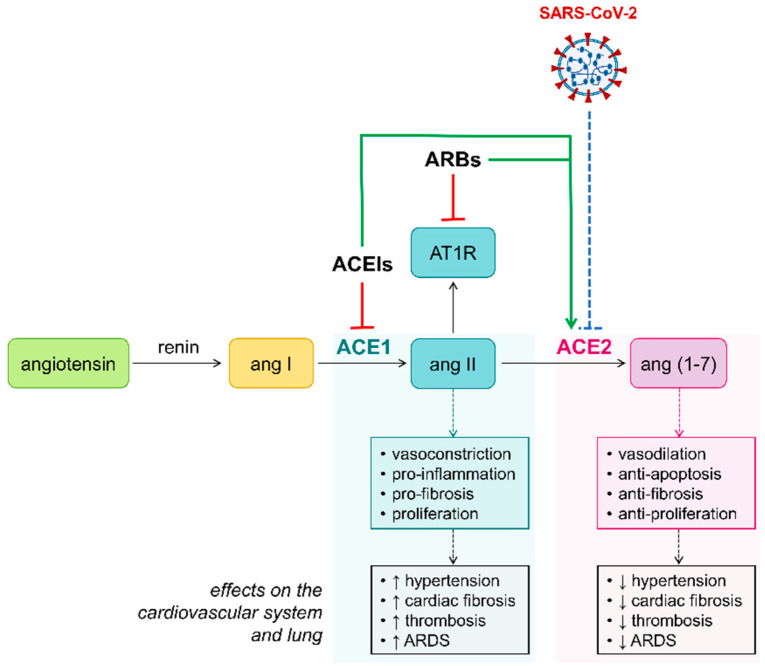
Schematic diagram showing the renin–angiotensin system (RAS) cascade and the effects on the cardiovascular system and lung. ACE1, angiotensin-converting enzyme 1; ACE2, angiotensin-converting enzyme 2; ang I, angiotensin I; ang II, angiotensin II; ang (1–7), angiotensin (1–7); AT1R, ang II type 1 receptor; ACEIs, angiotensin-converting enzyme inhibitors; ARBs, angiotensin receptor blockers; ARDS, acute respiratory distress syndrome. Green arrows indicate that ACEIs/ARBs increase ACE2 levels in the heart, therefore increasing the susceptibility of cardiac cells to SARS-CoV-2 infection [42,43]. Blue dotted hammerhead indicates the hypothetical effect of SARS-CoV-2 on ACE2 expression in lung and heart, which is based on the reported effect of SARS-CoV in the same body districts [45,46].

ACE2 is a key component of the renin–angiotensin system (RAS), which is a critical homeostasis regulation system of the human body, controlling blood pressure and the hydro-electrolyte balance [31]. ACE2 converts angiotensin II (ang II) to angiotensin 1–7 (ang 1–7), and it is a homolog of angiotensin converting enzyme 1 (ACE1), which, instead, is responsible for the conversion of angiotensin I (ang I) to ang II (Figure 1). The ACE2–ang (1–7) axis of RAS has the opposite effect to the ACE1–ang II axis [32]. Ang II has vasoconstrictive, pro-inflammatory, and pro-fibrotic abilities, therefore contributing to the increase of hypertension, cardiac fibrosis, thrombosis, and ARDS. On the contrary, ang (1–7) exhibits mild vasodilating, anti-fibrotic, and anti-proliferative effects, thus determining various cardio-protective actions, such as anti-thrombosis, anti-myocardial hypertrophy, anti-fibrosis, and anti-arrhythmia [33,34,35,36]. Beneficial actions of ang (1–7) have additionally been described in the lung, including reduction of lung inflammation, fibrosis, and pulmonary arterial hypertension [37,38,39,40].

As introduced above, it seems that there is a correlation between cardiovascular comorbidities and SARS-CoV-2 infection, although the mechanism of this association remains unclear. A possible explanation may lie in higher levels of ACE2 expression. Most patients with underlying CVD, such as hypertension, are treated with angiotensin-converting enzyme inhibitors (ACEIs) or angiotensin II receptor blocker (ARB) therapy [41]. These pharmacological treatments act by inhibiting ACE1 or blocking angiotensin II type 1 receptor (AT1R). Several studies have reported that both ACEIs and ARBs significantly increase mRNA expression of cardiac ACE2, thus stimulating its activity in generating ang (1–7), and finally contributing to significant cardiac and vascular protective effects [42,43,44]. Currently, we do not know whether ACEIs/ARBs can also influence ACE2 mRNA levels and protein activity in human lung tissues. At the same time, it has been speculated that patients with COVID-19 who are receiving these pharmacological agents may develop adverse outcomes, since ACE2 is a functional receptor for SARS-CoV-2. However, the role of ACE2 in viral infection is not yet fully understood, and its up-regulation may not be completely harmful. As demonstrated by an earlier study by Kuba and colleagues, the ACE2 expression in lung tissues was significantly down-regulated after SARS-CoV infection in mice, and this was accompanied by increased pulmonary vascular permeability and pulmonary edema [45]. Similarly, cardiac ACE2 down-regulation following SARS-CoV infection in mice was correlated with myocardial disfunction [46]. Considering the similarity with SARS-CoV, it has been speculated that SARS-CoV-2 infection might also down-regulate ACE2 expression in lung and heart, thus leading to the pathological processes of lung and cardiac injuries [27]. To date, no experimental or clinical data have evidenced that using ACEI/ARB therapy makes patients more susceptible to the virus. Therefore, several leading cardiovascular societies have strongly urged to not discontinue intake of RAS inhibitors in the event the patient develops COVID-19 [47,48].

## 3. Anti-Inflammatory and Immunomodulatory Properties of MSCs and MSC Secretome

MSCs are thought to prevent or reduce the cytokine storm in COVID-19 patients, owing to their powerful anti-inflammatory and immunomodulatory functions [8]. MSCs exert these effects by directly interacting with different cells of innate and adaptive immunity, including T cells, B cells, dendritic cells (DCs), macrophages, and natural killer cells, and by indirectly releasing many types of inflammatory mediators by paracrine secretion [49,50,51,52]. Many studies have described a differential regulation by MSCs on the different T cell subsets [53,54,55,56,57]. MSCs inhibit effector T (Teff) cell proliferation induced by mitogens or alloantigens by causing cell cycle arrest at the G1 phase [53,58]. Another explanation for this immunosuppressive capacity is the loss of CD25, the alpha-chain of the IL-2 receptor, which is cleaved from the activated T cell surface by MSC-secreted matrix metalloproteinases [59]. This leads to blockage of the IL-2 cytokine signaling pathway required for T cells activation, expansion, and differentiation. Interestingly, such T cell-suppressing properties of MSCs seem to require the presence of inflammatory cytokines in the microenvironment, which provoke the production of several T cell-attracting chemokines and inducible nitric oxide synthase (iNOS) from MSCs, so that T cells migrate into proximity of these cells [60]. At the same time, MSCs have been shown to induce the survival and expansion of regulatory T (Treg) cells, a subset of T cells involved in the suppression of proliferation and cytokine production by Teff cells [56]. Therefore, Treg cells foster the MSC-mediated immunosuppressive effect. In addition to directly interacting with T cells, MSCs also modulate the adaptive immune response by acting on antigen-presenting cells (APCs), such as DCs, monocytes, and macrophages, by shifting them to regulatory phenotypes characterized by T cell-suppressive properties [61,62].

The spectrum of regulatory factors secreted by MSCs is collectively defined as the MSC secretome, and include a complex array of soluble molecules, such as anti-inflammatory cytokines, angiogenic growth factors, antimicrobial peptides, and lipid mediators. A growing body of evidence nowadays suggests that some of these molecules are packaged into cell-secreted vesicles, known as extracellular vesicles (EVs) [63,64,65]. Besides apoptotic bodies, the two main types of EVs released by MSCs include exosomes and microvesicles (MVs). Exosomes (30–100 nm) are derived by fusion of multi-vesicular bodies with the plasma membrane, whereas MVs (100–1000 nm) are formed by cellular membrane budding, and contain cellular cytoplasm. All these EVs are released into the extracellular microenvironment, where they exert biological effects in a paracrine and endocrine manner, similarly to the soluble components. For this reason, a broader definition of MSCs secretome encompasses the entire spectrum of bioactive factors secreted by MSCs, which consists of both the soluble and the extravesicular elements. It has now been demonstrated that MSCs are also able to transfer functional mitochondria or mitochondrial DNA (mtDNA) to target cells, thus rescuing aerobic respiration in cells with non-healthy mitochondria or regulating T cell functions [66,67]. Following systemic injection, some MSCs accumulate in the lung, where they release these soluble mediators, potentially recovering the pulmonary microenvironment, protecting alveolar epithelial cells, and counteracting pulmonary fibrosis, thus resulting in a final improvement of lung function [8]. Moreover, distant injured organs, such as the cardiovascular system, can also benefit from them, by virtue of the secretory abilities of these cells.

To date, two studies have investigated the employment of MSCs in severely affected SARS-CoV-2 patients, with both reporting remarkable reversal of symptoms within a few days [10,68]. In one of these works, the levels of biochemical indicators of liver and myocardium damage (aspartic aminotransferase, creatine kinase activity, and myoglobin) returned to reference levels 4 days after MSC treatment [10]. The authors demonstrated that the cells expressed high levels of anti-inflammatory and angiogenic factors, such as transforming growth factor-beta (TGF-β), hepatocyte growth factor (HGF), leukemia inhibitory factor (LIF), fibroblast growth factor (FGF), vascular endothelial growth factor (VEGF), epidermal growth factor (EGF), brain-derived neurotrophic factor (BDNF), and nerve growth factor (NGF), further demonstrating their potent immunomodulatory abilities.

It has been reported that MSCs are generally resistant to viral infections compared to their differentiated progeny, probably due to intrinsic expression of IFN-stimulated genes (ISGs) [69]. Among these genes, those coding for proteins of the interferon-induced transmembrane (IFITM) family prevent viruses from traversing the lipid bilayer of the cell and accessing the cytoplasm, thus impairing viral infection [70]. These antiviral proteins limit infection in cultured cells by many viruses, including SARS-CoV, dengue virus, Ebola virus, influenza A virus, and West Nile virus. However, some studies have reported that human MSCs are permissive to other viruses, for example avian influenza viruses H1N1 and H9N5 and respiratory syncytial virus (RSV), losing vitality and compromising their immunomodulatory activities [71,72]. In the case of SARS-CoV-2, the advantage in using MSCs seems to be additionally related to the absence of ACE2 receptors on the cell surface, which precludes their recognition by the virus. Notably, in the study of Leng and coworkers, the cells remained negative for ACE2 also after transplantation in infected patients [10].

### 3.1. MSC-Derived EVs as a Therapeutic Pption for Critically Ill COVID-19 Patients

Although MSCs seem to be refractory to SARS-CoV-2 infection, in order to bypass the impact of viruses on MSCs, an interesting therapeutic strategy could consider the use of the MSC secretome. Among the bioactive factors released by MSCs, EVs, exosomes in particular, have gained remarkable interest in recent years because they enable more efficient communication and targeting than soluble molecules [73]. EVs, by virtue of their lipid bilayer membrane, better protect their molecular cargo of proteins and genetic material from environmental degradation (i.e., from trypsin or nuclease digestion) when compared to soluble molecules. Encapsulation within EVs may also facilitate delivery and targeting of these bioactive factors to distant recipient cells, mediated by binding of the EV surface proteins to cells that express appropriate receptors [74].

MSC-derived exosomes offer several advantages over traditional cell-based therapies. First, exosomes are considered safer than cells, because they are biocompatible, non-immunogenic, and lack the potential for endogenous tumors and emboli formation [75]. In addition, exosomes are physiologically more stable than cells, because their multiple membrane adhesion proteins allow for efficient binding in the target tissues during transplantation. Thanks to their resistant membrane, exosomes maintain their integrity during freezing and thawing procedures, making long-term storage without biological degradation possible [76]. In this context, a process has recently been proposed that combines ultrafiltration and lyophilization and is able to convert MSC secretome into a freeze-dried, ready-to-use powder [77,78]. The same research group also suggested the possibility to administer EVs by inhalation in the treatment of respiratory diseases [79]. This route of administration would benefit from lower invasiveness and pain, faster onset of action, and use of lower doses to achieve the same therapeutic effect when compared to oral or injection therapies. In this regard, a pilot clinical trial (NCT04276987) will be conducted in China for exploring the safety and efficiency of aerosol inhalation of MSC-derived exosomes in comparison to conventional treatment in 30 severe patients with COVID-19.

Another advantage of MSC-derived exosomes over whole-cell therapy is that, to improve their therapeutic potential, exosomes could potentially be modified with various types of cargos, including mRNA, microRNA (miRNA), and proteins, tailored to the disease process of interest [73]. In one pioneering work, exosomes incorporating the S protein have been explored as a novel vaccine approach against SARS-CoV infections [80]. The immunogenicity and efficacy of the S-containing exosomes were tested in mice, where they induced neutralizing antibody titers. Finally, from an economical point of view, MSC-derived exosome therapy might enable development of cheaper treatments other than the expansion and maintenance of individualized clonal cell populations [73]. This aspect is particularly important when a global pandemic has to be managed, as in the case of COVID-19. In Section 4, we provide an overview of the currently available evidence on the effects of MSC-derived exosomes in pre-clinical models of lung and heart injuries, which are the body districts most affected by SARS-CoV-2.

### 3.2. MSC-Derived EVs from Patients with Metabolic Disorders

Diabetes mellitus (DM) represents the most common inflammatory and chronic metabolic disorder worldwide, and continues to increase in number and significance—it is estimated that there will be 693 million persons with DM by 2045 [81]. Type 2 diabetes mellitus (T2DM) accounts for 90-95% of all cases of diabetes and results from a progressive defect in insulin production and insensitive response of the body to insulin [82]. Accumulating evidence shows that such a state of insulin resistance (IR) is closely related with obesity [83]. Obesity, mainly visceral adiposity, is, indeed, one of the most important comorbidities in diabetic patients.

People with diabetes have a higher overall risk of infections that result from compromised innate cell-mediated immunity; impaired phagocytosis by neutrophils, macrophages, and monocytes; and impaired neutrophil chemotaxis and bactericidal activity [84]. Regarding COVID-19, it is currently unknown whether patients with diabetes have a higher susceptibility to the virus; nonetheless, there is evidence of higher risk for both infection and disease severity [85].

As stated above, there is growing interest in the use of MSC-derived EVs as a therapeutic tool for the management of several diseases. However, because EV cargo usually reflects parent cell characteristics, and these are influenced by the metabolic state of source cells, it is reasonable to consider the risks associated with the employment of MSC-derived EVs from patients with coexisting metabolic disorders such as T2DM. In effect, clinical studies have found differences in the number and composition of EVs isolated from the adipose tissue of obese patients and from animal models of obesity [86,87,88,89]. For example, MSC EVs isolated from a swine model of metabolic disorder were found to be enriched with mRNAs associated with inflammation, such as those coding for the integrin family proteins, or proteins of the FGF signaling [87]. These MSC-derived EVs also showed a distinctive miRNAs cargo, being enriched in miRNA-targeting genes involved in the development of metabolic disease and its complications, including diabetes, obesity, and insulin signaling [88]. Apart from influencing the mRNA and miRNA content, metabolic disorder also alters packaging of proteins into porcine MSC-derived EVs, promoting the inclusion of pro-inflammatory proteins, such as those involved in acute inflammatory response, cytokine production, and leukocyte transendothelial migration [86]. The limitations of these works reside in the small sample size and short duration of metabolic disease compared to the human condition; therefore, further studies would be needed to draw clear conclusions. However, in humans also, analysis of adipose tissue-derived EVs demonstrated that obesity alters their cargo of mRNAs, miRNAs, and proteins [90]. In particular, the differentially expressed miRNAs contained in the isolated EVs stimulated up-regulation of Wnt/β-catenin and TGF-β signaling pathways, which are related to inflammation, into A549 lung epithelial cells.

Overall, these observations suggest that diabetes and metabolic disorders might alter the MSC-derived EV cargo, which in turn might compromise their anti-inflammatory and immunodulatory potential both in the endogenous microenvironment and after autologous transplantation.

## 4. Pre-Clinical Evidence on the Use of MSC-Derived Exosomes in Animal Models of Lung and Heart Injuries

### 4.1. MSC-Derived Exosomes and Respiratory Lung Injuries

Acute lung injury (ALI) and ARDS are major causes of respiratory failure in critically ill ventilated patients, with an estimated 60-day mortality rate of 32% [91]. ARDS is also one of the most common complications in severely affected COVID-19 patients. The term ARDS is often used interchangeably with ALI; nevertheless, ARDS should be reserved for the most severe form of the disease [92]. Bacterial or viral infections are the most common causes of ALI and ARDS; however, they can also be initiated by aspiration of gastric contents, toxic inhalation, lung contusion, or trauma [92]. The acute phase (the first 1–6 days) of the diseases is characterized by injury to both the pulmonary endothelium and the alveolar epithelium, the two barriers forming the alveolar–capillary barrier. In healthy lung microvessels, the pulmonary endothelium is maintained by vascular endothelial cadherin (VE-cadherin), an endothelial-specific adherens junction protein, whereas the alveolar epithelial barrier has E-cadherin junctions and is substantially less permeable than the endothelial counterpart [93,94]. During lung injury, VE-cadherin bonds are destabilized by increased expression of thrombin, TNF-α, VEGF, and signals from leukocytes. At the same time, E-cadherin epithelial junctions are disrupted by neutrophil migration, which causes injury, apoptosis, and membrane denudation. This ultimately results in increased epithelial permeability, leading to accumulation of protein-rich edema fluid in the alveoli, and in turn to an impairment in gas exchange and to hypoxemia [95]. Dysregulated immune activation has also been implicated in the pathogenesis of ALI/ARDS. In the air space, macrophages release pro-inflammatory cytokines and chemokines, which act locally to stimulate chemotaxis and activate neutrophils. Activation of neutrophils leads to the release of numerous cytotoxic products, such as reactive oxygen species, cationic peptides, eicosanoids, and proteolytic enzymes, which may further damage the alveolar epithelium [96].

Resolution of ALI/ARDS aims at removing alveolar edema fluid, repairing the epithelial and endothelial barriers, and removing inflammatory cells and exudate from the air spaces [95]. To date, management of ALI/ARDS includes lung protective ventilation, prone positioning, neuromuscular blockade, and extracorporeal membrane oxygenation. Mechanical ventilation represents the mainstay treatment in ALI/ARDS, and consists in the application of positive-end expiratory pressure for optimizing arterial oxygenation. It has been evidenced that ventilation with a low tidal volume (6 mL/kg) gives better results when compared to traditional tidal volume (12 mL/kg) [97]. Indeed, the use of lower tidal volumes during ventilation may reduce injurious lung stretch and the release of inflammatory mediators. Prone positioning enhances arterial oxygenation by improving alveolar ventilation/perfusion matching. Nevertheless, this treatment should be used with caution and should be reserved for patients with critical hypoxemia, since it does not improve survival or decrease the duration of lung ventilation. All these therapeutic options remain primarily supportive; on the other hand, alternative treatments with glucocorticoids, surfactants, inhaled nitric oxide, antioxidants, protease inhibitors, or other anti-inflammatory agents had proven unsuccessful in reducing mortality or improving ALI/ARDS outcomes [96].

In terms of promising novel strategies, MSC-based approaches have been explored for the management of ALI/ARDS. The benefit of MSC therapy appears to be related to a decrease in pro-inflammatory cytokines and to an increase in anti-inflammatory cytokines, particularly IL-10 [98]. MSCs release prostaglandin E_2_, which in turn stimulates secretion of IL-10 by monocytes and alveolar macrophages [99]. Moreover, administration of MSCs seems to be effective in normalizing lung endothelial and epithelial permeability to protein, as well as in reducing pulmonary edema and increasing the rate of alveolar fluid clearance [100].

Recently, MSC-derived exosomes have been demonstrated to have comparable and even greater effects than cells themselves in improving inflammation and injury in a variety of pre-clinical lung disease models, including ALI/ARDS (Table 1). For the completeness of information, we have to specify that some of these works take into account the entire spectrum of EVs that, in addition to exosomes, also includes MVs. This is because as of yet there are no standardized methods for isolation, quantification, and characterization of EVs, or for discriminating MVs and exosomes. Consequently, in the majority of these pre-clinical studies, EVs, exosomes, and MVs are collectively referred to as EVs.

MSC-derived EVs have been proven to be beneficial in both bacteria- and virus-induced ALI/ARDS. A large number of studies have employed an endotoxin-mediated in vivo model to investigate the effects of MSC-derived EVs for ALI/ARDS. In one of the first works, ALI was induced in C57BL/mice using the intratracheal (IT) instillation of endotoxin (4 mg/kg) from *Escherichia coli* (*E. coli*) [101]. MVs were isolated from the conditioned medium of human bone marrow-derived MSCs with two sequential ultracentrifugations at 100,000× *g* for 1 h. Then, 30 uL of MVs, corresponding to the vesicles released by 3 × 10^6^ MSCs, were administrated intratracheally or intravenously in mice. After 48 h, MSC-derived MVs reduced lung inflammation and reduced edema to the same levels as MSCs themselves, which were used as a positive control. Furthermore, MVs also decreased the influx of neutrophils and MIP-2 levels in the alveolar fluid, indicating a reduction in inflammation. Surprisingly, the therapeutic effects of the MVs were comparable, regardless of route of administration. The authors suggested that the mechanism underlying the therapeutic effect of MVs might be in part mediated by the transfer of keratinocyte growth factor (KGF) mRNA into the injured alveolar epithelium, with subsequent expression of the protein. KGF is an epithelial-specific growth factor released from MSCs, which has been shown to reduce lung edema and inflammation in various ALI models [102,103,104]. In the same study, the effect of MVs was additionally evaluated in RAW 264.7 cells, a mouse macrophage cell line. Treatment with 30 μL of MSC-derived MVs to endotoxin-stimulated RAW 264.7 cells reduced the levels of TNF-α and MIP-2, and concomitantly increased the production of the anti-inflammatory cytokine IL-10 at 6, 12, and 24 h compared with endotoxin-stimulated mouse macrophages [101]. In the work of Tang and colleagues, ALI was induced in C57BL/mice by the instillation of lipopolysaccharide (LPS) from *Pseudomonas aeruginosa* at 4 mg/kg intratracheally [105]. MVs released from human bone marrow MSCs were isolated by two sequential ultracentrifugations at 100,000× *g* for 1 h, then intratracheally administrated in endotoxin-injured mice. IT administration of MSC-derived MVs improved the lung inflammation induced by LPS in mice, including the influx of white blood cells and neutrophils, and MIP-2 secretion. In that study, the authors found that the transfer of angiopoietin-1 (Ang-1) mRNA by MVs was essential for the reduction of inflammation and the restoration of alveolar-capillary barrier. Ang-1 plays a key role in vascular stabilization, since it reduces endothelial permeability and suppresses leukocyte–endothelium interactions [106]. Furthermore, MSC-derived MVs showed immunomodulatory effects on RAW 264.7 cells in vitro by inhibiting TNF-α mRNA production and promoting the mRNA levels of IL-10 after 3 h [105]. Collectively, these two studies suggest that the beneficial immunomodulatory effect of MSC-derived MVs in ALI is strongly dependent on KGF and Ang-1 mRNA transfer into injured endothelial cells.

It has been demonstrated that, apart from mRNAs, the therapeutic effect of EVs is also mediated by the transfer of functional mitochondria to target cells. MSCs have been reported to naturally transfer mitochondria to recipient cells through different mechanisms—incorporated within EVs, via cell-to-cell contact through tunneling nanotubes, or through direct release of naked mitochondria into the extracellular microenvironment [107,108]. In the work of Phinney and colleagues, transmission electron microscopy images evidenced structures consistent with the morphology of mitochondria inside MVs over 100 nm in size, previously isolated from the conditioned medium of human MSCs after centrifugation at 10,000× *g* for 1 h [109]. The authors found that these mitochondria were loaded in the cytoplasm into LC3-containing MVs, which migrated towards the cell periphery and were incorporated into outward budding blebs in the plasma membrane. The MSC-derived MVs contained functionally active mitochondria that were taken up by macrophages and resulted in improved bioenergetics after oxidative stress increment. In particular, the transfer of human MSC-derived mitochondria involved fusion with mitochondria inside macrophages, suggesting that the mitochondrial membrane was not collapsed. The same study also confirmed that mitochondria were not packaged within exosomes; rather, exosomes were able to deliver mtDNA, which in mammals has an average size under 100 nm [110]. In the context of lung injury, the group of Morrison and colleagues revealed that MSC-derived EVs protected mice against LPS-induced ALI by altering alveolar macrophage (AM) polarization from the pro-inflammatory M1 phenotype towards the M2 anti-inflammatory phenotype [111]. In detail, EVs were obtained from human bone marrow MSCs after ultracentrifugation at 100,000× *g* for 2 h. These were used for pre-treating AM, which were then intranasally administrated to LPS-injured mice. The MSC-derived EVs increased phagocytic activity by macrophages and reduced their secretion of TNF-α and IL-8, two major pro-inflammatory cytokines related to ARDS severity [112,113]. The transfer of functional mitochondria contained in EVs, associated with the promotion of oxidative phosphorylation, was supposed to be the mechanism responsible for the observed effects in macrophages [111].

The work of Monsel and colleagues was the first to evaluate the effect of MSC-derived MVs in an infectious ALI model [114]. Indeed, the authors instilled live *E. coli* bacteria into the trachea; then, they intravenously administrated 90 uL of MVs, corresponding to the vesicles released by 9 × 10^6^ human bone marrow MSCs. MV injection improved survival and reduced the bacterial load, as well as the influx of white blood cells, neutrophils, and MIP-2 levels, in the injured alveolus of C57BL/mice. The authors reported that the effect was in part mediated by KGF mRNA shuttled by the vesicles into target cells, as described in their previous study [101]. In addition to testing in mice, the effect of MSC-derived MVs was also investigated in human monocytes and AT2 cells. MV treatment increased the percentage of phagocytosis of human monocytes against *E. coli* bacteria, thus reducing the bacterial count, and decreased TNF-α secretion. Furthermore, MVs showed a beneficial effect on injured human AT2 cell metabolism through the restoration of intracellular ATP levels to control levels. In these primary in vitro cultures, the uptake of MVs was mediated by CD44, which was essential for the observed therapeutic effects. CD44 is the hyaluronic acid receptor expressed in almost every cell type including MSCs [51,115]. The results of this work suggested that MVs, similarly to their parent cells, act through different mechanisms on the basis of anti-inflammatory, anti-microbial, and metabolomic effects.

MSC-derived EVs have also shown reparative properties on microvascular endothelial and epithelial cells, which are often severely injured in the lung during ALI, and are associated with increased mortality in ARDS patients. Hu and coworkers investigated the effects of MVs isolated from human bone marrow MSCs on human lung microvascular endothelial cells (HLMVECs) in vitro [116]. The cells were injured by cytomix, a mixture of the most biologically active cytokines found in ALI pulmonary edema fluid (IL-1β, TNF-α, and IFN-γ at 50 ng/mL), and simultaneously exposed to increasing doses (30 or 60 uL) of MVs using a transwell co-culture system [117]. Administration of MSC-derived MVs restored protein permeability of HLMVECs by preventing the reorganization of cytoskeleton protein F-actin into “actin stress fibers” and the loss of tight and adherens junction proteins (zonula occludens-1 and VE-cadherin, respectively) following inflammatory injury. The internalization of MVs via cluster of differentiation (CD)44 receptor, as well as the subsequent transfer of Ang-1 mRNA into injured HLMVECs, were required for the observed therapeutic effects.

The study of Khatri and colleagues is interesting because ARDS was induced in pigs after infection with a mixed swine (H3N2, H1N1) and avian (H9N5, H7N2) influenza viruses (SwIV) [118]. Pigs are often used as large animal pre-clinical models for several human diseases, including respiratory diseases, due to their close similarity in anatomy, physiology, and immunology to humans [119]. In addition, influenza virus pathogenesis and clinical signs are similar to those observed in humans. In that work, EVs (80 μg/kg) isolated from swine bone marrow MSCs with two ultracentrifugation steps at 25,000 rpm for 70 min were intratracheally administrated in pigs 12 h after SwIV inoculation. MSC-derived EVs were found to inhibit influenza virus replication and shedding in pigs 3 days post-infection. As in other studies, EVs also modulated inflammatory cytokine and chemokine production in the lungs, as demonstrated by reduction in TNF-α and CXCL10 protein levels, and increase in IL-10 protein levels. Unfortunately, there are not yet pre-clinical data on the effects of MSC-derived EV administration in models of coronavirus respiratory infection, mostly due to the lack of an established animal model [120].

**Figure 2:**
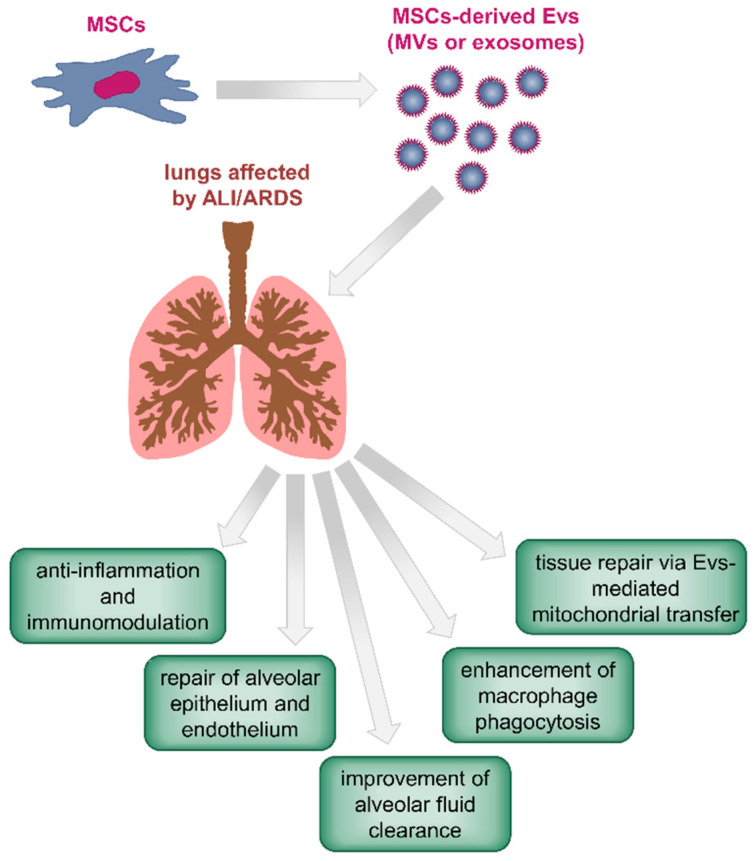
Schematic diagram showing the proposed mechanisms of the beneficial therapeutic effects of MSC-derived EVs (MVs or exosomes) in acute lung injury (ALI)- or acute distress respiratory syndrome (ARDS)-affected lungs. These are based on immunomodulation and anti-inflammatory functions of MSC EVs [101,105,114,118], reduction of the permeability of alveolar epithelium and endothelium [116], improvement of alveolar fluid clearance [114], enhancement of macrophage phagocytosis [111], and tissue repair through direct mitochondrial transfer with host cells [111].

From the studies discussed above, it emerged that the rationale for using MSC-derived exosomes, MVs, or EVs in ALI/ARDS is based on several processes, many of which are shared with those identified in the parent MSCs. These include immunomodulation and anti-inflammatory properties on host tissue, reduction of the permeability of alveolar epithelium and endothelium, improvement of alveolar fluid clearance, enhancement of macrophage phagocytosis, and tissue repair through direct mitochondrial transfer with host cells (Figure 2).

Apart from ALI/ARDS, there have been several investigations using MSC-derived EVs as a potential therapy in the management of other lung diseases, such as bronchopulmonary dysplasia, pulmonary arterial hypertension, idiopathic pulmonary fibrosis, and asthma, which have been recently revised in Worthington [121] and Behnke [122]. Moreover, in these lung diseases, the most common effects of MSC-derived EVs were decreased inflammation and restoration of the lung architecture, achieved through the reduction of fibrosis and increase of vascularization and alveolarization.

### 4.2. MSC-Derived Exosomes and Heart Injuries

Acute myocardial injury has been described as the most common cardiovascular complication in COVID-19 patients [13]. Myocardial injury is defined as an elevation in serum levels of high-sensitive cardiac troponin (cTn) above the 99th percentile upper reference limit, although over the years it has also been identified through an increase in different cardiac enzymes and/or electrocardiographic abnormalities [123]. The injury is considered acute if there is a dynamic rise and/or fall of cTn values. When acute myocardial injury is caused by myocardial ischemia, it is designated as acute myocardial infarction (AMI). On the contrary, myocardial injury not related to ischemic events may arise secondary to many cardiac conditions, such as myocarditis [123].

**Figure 3:**
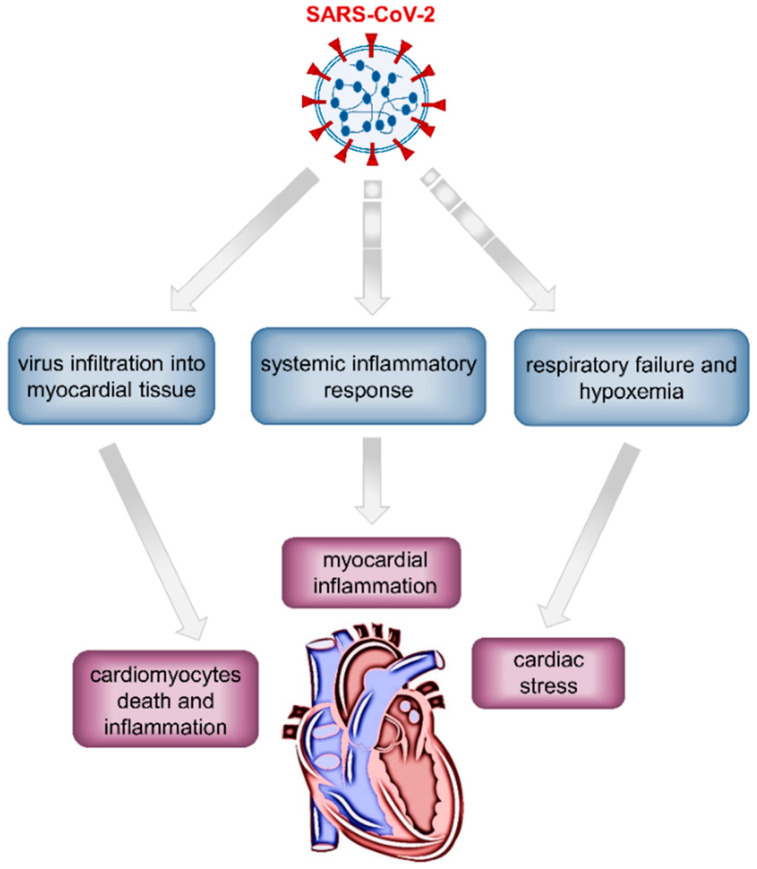
Schematic diagram showing the proposed mechanisms of the severe acute respiratory syndrome coronavirus 2 (SARS-CoV-2)-mediated myocardial injury. The solid arrow indicates a direct mechanism involving virus infiltration into the myocardium, whereas dotted arrows represent indirect mechanisms based on systemic inflammation, respiratory dysfunction, and hypoxemia.

Analyzing several reports from China, a considerable proportion of patients (12–27.8%) presented elevated cTn levels, and most of them required ICUs and showed higher in-hospital mortality [7,124,125,126]. The mechanisms of myocardial injury are not well established but likely involve direct or indirect processes and/or their combination (Figure 3). Myocardial infection by SARS-CoV-2 resulting in cardiomyocyte death and inflammation has been proposed as a possible direct mechanism, although, to date, there are no data demonstrating the presence of SARS-CoV-2 within myocardial tissue [127]. Nevertheless, a previous autopsy study in patients who died from SARS identified the viral RNA in 35% of the post-mortem human heart samples, providing evidence for direct myocardial injury by the virus [46]. In addition, patients carrying SARS-CoV in their hearts died considerably earlier, suggesting that viral infiltration in the myocardium was associated with a more aggressive course of illness. Systemic inflammatory response or respiratory failure and hypoxemia can represent indirect mechanisms leading to increased cardiac stress and myocardial inflammation [127,128]. In a couple of studies, biopsies taken from heart tissue of COVID-19 patients evidenced mononuclear inflammatory infiltrates, mainly associated with regions of cardiomyocyte necrosis, which identifies myocarditis according to Dallas criteria [129,130,131]. Nevertheless, acute lymphocyte infiltrates were not observed in the myocardium of SARS-CoV-2-infected patient autopsy.

Other aspects of COVID-19 in cardiac involvement include blood pressure abnormalities and arrhythmias, ranging from tachycardia and bradycardia to asystole [127]. Very recently, it has also been suggested that there is a link between SARS-CoV-2 infection and Kawasaki disease (KD), especially in pediatric patients [132]. Although KD is a disease of unknown etiology, infections are considered to be one of the predisposing factors [133]. The disease predominantly affects children under 5 years of age and causes inflammation in the walls of medium-sized arteries, primarily the coronary arteries, those that supply blood to the heart muscle.

Consequences of AMI are loss of cardiomyocytes and adverse remodeling of the extracellular matrix, which contribute to the reduction of pumping of the heart and further heart failure. Nowadays, the best therapeutic strategy for reducing AMI is timely and effective myocardial reperfusion. However, this treatment induces oxidative stress and inflammation, thus leading to further cardiomyocyte death, myocardial remodeling, and decreased cardiac function, a phenomenon known as myocardial reperfusion injury [134].

Over the last years, management of AMI using stem cell therapy was found to prevent myocardial cell apoptosis, promote local neoangiogenesis, and reduce the local inflammatory response [135,136,137]. Similarly to what was described above for lung injuries, the beneficial effect of stem cells seems to be largely attributable to the secreted EVs. Since the first description of the therapeutic potential of MSC-derived exosomes in a mouse model of myocardial ischemia/reperfusion (I/R) injury in 2010, several studies have subsequently reported cardio-protective effects of MSC-derived EVs in AMI animal models (Table 2) [138]. In one of these works, a single intravenous (IV) injection of MSC-derived exosomes in a mouse AMI model led to decreased infarct size, enhanced nicotinamide adenine dinucleotide (reduced form) (NADH) and ATP levels, and reduced oxidative stress, which are hallmarks of reperfusion injury [139]. All these events seemed to be associated with the exosome-mediated activation of the pro-survival phosphoinositide 3-kinase/protein kinase B (PI3K/Akt) signaling pathway, which resulted in an enhancement of myocardial viability and prevented adverse remodeling after myocardial I/R injury. Importantly, intact but not lysed exosomes were responsible for the improved cardiac function after AMI induction.

Another important mechanism by which MSC-derived EVs contribute to ischemic myocardial repair is through stimulation of neovascularization, as shown in the work of Bian and colleagues [140]. Neovascularization refers to processes, such as vasculogenesis, angiogenesis, and arteriogenesis, that are associated with migration and proliferation of endothelial cells. In line with these findings, Ma and coworkers also demonstrated that exosomes isolated from Akt-transfected MSCs accelerated angiogenesis in a rat myocardial infarction model [141]. The authors suggested that platelet-derived growth factor D (PDGF-D), which was enriched in MSC-derived vesicles, was mainly responsible for the Akt exosome-mediated improvement of myocardial repair. A more recent study by Xuan and colleagues identified Notch1 as a potent modulator of angiogenesis and cardiomyocyte proliferation into ischemic mice hearts following coronary heart ligation [142]. The role of Notch1 signaling in inducing cardiac angiogenesis during ischemia and enhancing survival of cardiac cells is well established [143]. The injection of MSC-derived EVs over-expressing Notch1 intracellular domain (NICD) in ischemic myocardium led to decreased infarct size, improved cardiac function, and increased arteriole density in the peri-infarct area, 1 month after AMI [142]. Moreover, Teng and coworkers indicated that the beneficial effect of MSC-derived exosomes on infarcted rat hearts is mainly dependent on their angiogenesis-promoting activity [144]. In their study, the authors proved that exosomes also act by restraining the inflammatory response. In agreement with the results of Arslan and colleagues, they also demonstrated that fresh exosomes achieved a better therapeutic effect with respect to frozen exosome preparations.

Several studies agree that reduced fibrosis and apoptosis of myocardial cells are other important effects of the EV-mediated ischemic cardiac repair [145,146,147,148]. In particular, Zhao and coworkers showed that human umbilical cord MSC-derived exosomes improved cardiac function and reduced cardiac fibrosis by preventing cardiomyocyte apoptosis and promoting cell proliferation in the border zone of infarcted rats [146]. The effect mediated by exosomes was attributed to the up-regulation of the anti-apoptotic protein B cell lymphoma 2 (Bcl-2) in the myocardial cells. Other works have proposed that specific functional miRNAs contained into EVs and shuttled to target injured cells are primarily responsible for the beneficial effects. For example, Feng and colleagues found that miR-22 was up-regulated in MSC-derived EVs, and it possibly reduced cardiac apoptosis and fibrosis in an AMI mouse model via inhibition of methyl cytosine-phosphate-guanine (CpG)-binding protein 2 (Mecp2) expression [145]. In that study, the authors isolated EVs from MSCs subjected to ischemic pre-conditioning, which is an effective approach to potentiate survival and regeneration of these cells in an ischemic environment. Yu and coworkers identified miR-19a as the molecular mediator able to restore cardiac function and reduce infarct size in a rat model of AMI [147]. The cardio-protective role of miR-19a was mediated by down-regulation of target genes, phosphatase and tensin homolog (PTEN), and Bcl-2 interacting mediator of cell death (BIM) in cardiomyocytes and subsequent activation of the Akt and extracellular signal-regulated kinase (ERK) signaling pathways. In their study, exosomes were isolated from MSCs over-expressing GATA binding protein 4 (GATA-4), a transcription factor able to regulate miRNA expression in MSCs and increase their survival in an ischemic environment [149]. Apart from observing reduced cardiac fibrosis and reduced inflammation in infarcted rat hearts 7 days after exosome injection, Shao and colleagues identified a panel of miRNAs, which were similarly up- or down-regulated in MSCs and the derived exosomes [148]. On the other hand, other miRNAs, such as miR-21 and miR-15, resulted as being differentially expressed between exosomes and MSCs, with this potentially explaining why MSC-derived exosomes demonstrated superior beneficial effects when compared with treatment with their parent cells.

Several studies have demonstrated that autophagy also has an important role in mediating the therapeutic effects of MSC-derived exosomes. Autophagy is known to be an important mechanism in cardio-protection, and dysregulated autophagy is associated with a variety of CVD [150]. In particular, it has been demonstrated that exosomes reduce apoptosis and the myocardial infarct size, as well as improve cardiac function by inducing cardiomyocyte autophagy both in vitro and in vivo [151,152].

**Figure 4:**
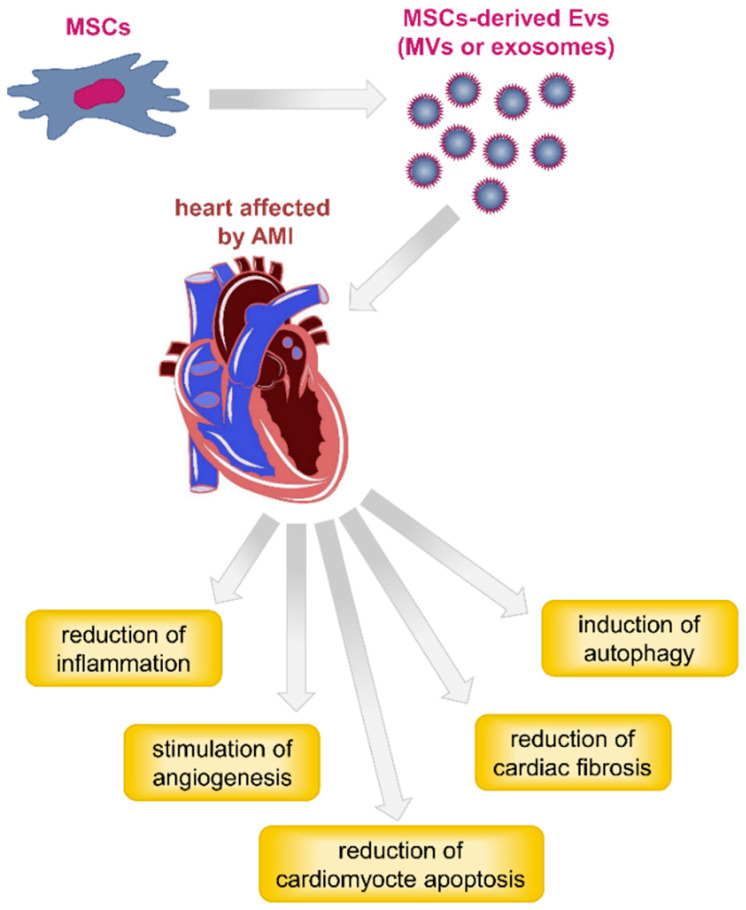
Schematic diagram showing the beneficial therapeutic effects of MSC-derived EVs (MVs or exosomes) in heart affected by acute myocardial infarction (AMI). These are reduction of the inflammatory response [139,144,148], reduction of cardiac fibrosis, reduction of cardiomyocyte apoptosis [145,146,148,149,151], promotion of angiogenesis [140,141,142,144], and induction of cardiomyocytes autophagy [151].

Collectively, the described studies documented reduction in infarct size with improved recovery of cardiac function, reduction of fibrosis and apoptosis, stimulation of angiogenesis, and decreased infiltration of macrophages and other immune cells into the injured heart regions following treatment with MSC-derived EVs (Figure 4). When comparing the properties of exosomes recovered from different MSCs sources, those isolated from adipose tissue samples exhibited the strongest cardio-protective effects [153].

## 5. Conclusions and Future Perspectives

The recent coronavirus COVID-19 global pandemic has driven the need for novel urgent therapies. MSCs and their derivatives are being evaluated for the treatment of a number of diseases that currently have limited or no therapeutic options. MSC-derived EVs (exosomes and MVs) have recently attracted great attention because, similarly to their parent cells, they possess strong anti-inflammatory, immunomodulatory, and pro-angiogenic abilities, just to name a few. However, compared to MSCs themselves, EVs hold many biological and technological advantages. EV administration is considered safer than MSC transplantation, lacking some of their negative side-effects, and they are more stable than MSCs themselves, allowing for easier handling and storage. Over the last years, a plenty of pre-clinical studies in animal models have demonstrated that the administration of MSC-derived EVs significantly reduced lung inflammation and pathological impairment subsequent to different types of lung injury, as well as resulted in improved cardiac function after acute myocardial injury. However, several challenges still need to be overcome to make the transition from animal models to humans possible. For example, standardized techniques for isolation, characterization, and quantification, as well as criteria for establishing dose, quality control, and storage conditions of MSC-derived EVs, are required before these can be advanced to the clinic. To date, it is difficult to compare and analyze studies employing MSC-derived EVs since there is a large degree of heterogeneity in EV preparations, and because MSC-derived EVs differ depending on tissues and donors from which the cells are isolated.

Regarding COVID-19, the lack of an established animal model of coronavirus-induced lung injury requires a more prudent and careful use of MSC-derived EVs. In this context, a significant issue is to establish under what circumstances and with what criteria to administer MSC-derived EVs. For example, which population among COVID-19 patients to target and when to start EV administration. Moreover, there remains the challenge to clarify the optimal route of EV administration that, in the case of lung diseases, mostly occurs through IT instillation or IV injection, although the possibility of EV inhalation has recently been explored. To date, no studies have investigated the biodistribution and the in vivo metabolic fate of EVs following IT instillation. On the other hand, systemic IV injection has been shown to deliver EVs primarily to the spleen and liver, then to the gastrointestinal tract and lungs, followed by renal and hepatic clearance in mice [138,154]. Apart from the administration route, another major issue concerns the optimal EV therapeutic dose. Considering that the average therapeutic dose of MSCs for treating lung injuries is 10 × 10^6^ cells/kg per body weight, the amount of cells required to generate enough EVs to achieve the equivalent effect of MSCs is generally 5–10 times higher [155]. This necessitates large scale production of MSC-derived EVs. Although this could be implemented with the use of bioreactors for MSC expansion, different bioreactor culture conditions would result in alterations of EV content, which in turn may impact on the therapeutic efficacy. Another challenge to consider for the administration of MSC-derived EVs to COVID-19 patients is the need to manufacture a safe and reproducible therapeutic product. Since the production of EVs requires the use of living cells, these have to be cultured under good manufacturing practice (GMP)-compliant procedures to preserve the quality and safety standards criteria. Therefore, EV production must follow the same rigorous scientific and ethical guidelines that apply to MSCs, and any therapy based on MSC-derived EVs needs to be approved by the national regulatory agencies to demonstrate its safety and efficacy. In light of these observations, in our opinion, the use of MSC EVs could be contemplated to treat critically ill patients with ARDS requiring mechanical ventilation or ICU support, or patients with recognized risk factors, such as pre-existing CVD, or cardiovascular complications, for whom standard therapeutic approaches have not proven resolutive.

## Figures and Tables

**Table 1:** Successful use of mesenchymal stem cell (MSC)-derived extracellular vesicles (EVs) (microvesicles (MVs) or exosomes) in lung injury.

Disease	Model	EV ^1^ Origin	EV Administration Route and Dosage	Main Findings	Reference
ALI ^2^	IT ^3^ instillation of *Escherichia coli* endotoxin (4 mg/kg) in C57BL/mice	MSCs from human bone marrow	IT or IV ^4^ administration of 30 μL (3 × 10^6^ cell equivalents) of MVs ^5^ simultaneously to endotoxin inoculation	reduction of extravascular lung water, pulmonary edema, and lung protein permeability after 48 h compared to endotoxin-injured mice; reduction of neutrophil infiltration and MIP-2 ^6^ levels; essential role of KGF ^7^ mRNA transported by MVs into injured cells; reduction of TNF-α ^8^ and MIP-2 levels and increase in IL-10 ^9^ production in endotoxin-stimulated RAW 264.7 cells at 6, 12, and 24 h;	[101]
ALI	IT instillation of LPS ^10^ (4 mg/kg) from *Pseudomonas aeruginosa* in C57BL/mice	MSCs from human bone marrow	IT administration of 30 μL (3 × 10^6^ cell equivalents) of MVs simultaneously to endotoxin inoculation	restoration of pulmonary capillary permeability; reduction of lung inflammation in terms of white blood cells and neutrophils influx, and MIP-2 levels at 48 h; essential role of Ang-1 ^11^ mRNA transported by MVs into injured cells; reduction of TNF-α mRNA and increase in IL-10 mRNA levels in endotoxin-stimulated RAW 264.7 cells at 3 h;	[105]
ALI	intranasal instillation of LPS (20 mg/kg) from *Escherichia coli* in C57BL/mice	MSCs from human bone marrow	intranasal administration of AM ^12^ (2.5 × 10^5^ cells/mouse) pre-treated ex vivo with MSC-derived EVs 4 h after endotoxin inoculation	transfer of functional mitochondria through EVs, which resulted in oxidative phosphorylation, which in turn led to enhanced phagocytosis and TNF-α and IL-8 ^13^ secretion by macrophages;	[111]
ALI	IT instillation of *Escherichia coli* K1 strain (2 or 3 × 10^6^ cfu ^14^) in C57BL/mice	MSCs from human bone marrow	IV administration of 90 uL (9 × 10^6^ cell equivalents) of MVs 4 h after bacterial inoculation	reduction of the bacterial load in the alveolar fluid at 18 h; reduction of the influx of white blood cells and neutrophils at 24 h; reduction in MIP-2 levels in the alveolar fluid at 18 and 24 h in vivo; increase of bacterial clearance and decrease of TNF-α secretion by LPS-primed human monocyte cultures at 24 h; restoration of ATP levels by injured human AT2 ^15^ cell cultures at 48 h; essential role of CD44 for MVs internalization;	[114]
ALI	HLMVECs ^16^ injured by cytomix (50 ng/mL of IL-1β, TNF-α, and IFN-γ)	MSCs from human bone marrow	30 or 60 uL of MVs (10 uL corresponding to MVs released by 1 × 10^6^ MSCs) simultaneously to cytomix treatment	prevention of the reorganization of cytoskeleton protein F-actin into “actin stress fibers”; restoration of the location of the tight junction protein zonula occludens-1 and adherens junction protein VE-cadherin in injured HLMVECs; essential role of CD44 for MVs internalization; essential role of Ang-1 mRNA transported by MVs into injured cells;	[116]
ARDS ^17^	intranasal inoculation of swine influenza virus (5 × 10^6^ TCID50 ^18^/pig) in White-Duroc crossbred pigs	MSCs from swine bone marrow	IT administration of EVs (80 µg/kg) 12 h after viral infection	inhibition of influenza virus replication and shedding; reduction in TNF-α and CXCL10 protein levels, and increase in IL-10 protein levels 3 days post-infection.	[118]

^1^ extracellular vesicles; ^2^ acute lung injury; ^3^ intratracheal; ^4^ intravenous; ^5^ microvesicles; ^6^ macrophage inflammatory protein-2; ^7^ keratinocyte growth factor; ^8^ tumor necrosis factor-alpha; ^9^ interleukin-10; ^10^ lipopolysaccharide; ^11^ angiopoietin-1; ^12^ alveolar macrophages; ^13^ interleukin-8; ^14^ colony forming units; ^15^ alveolar type II; ^16^ human lung microvascular endothelial cells; ^17^ acute distress respiratory syndrome; ^18^ median tissue culture infectious dose.

**Table 2:** Successful use of MSC-derived EVs (MVs or exosomes) in heart injury.

Disease	Model	EVs Origin	EVs Administration Route and Dosage	Main Findings	Reference
I/R ^1^ injury	30 min ischemia through ligation of LCA ^2^ in C57BL/6J mice followed by 24 h reperfusion	MSCs from HuES9.E1 cells	IV ^3^ injection of 0.4 µg/mL of exosomes, 5 min before reperfusion	reduction of the infarct size by 45% in vivo and ex vivo in Langendorff model; increase in ATP and NADH levels, and decrease in oxidative stress within 1 h of reperfusion; increase in Akt and GSK3 ^4^ phosphorylation, and reduction in phosphorylation of pro-apoptotic c-JNK ^5^ within 1 h of reperfusion; reduction in neutrophil and macrophage infiltration at 1 and 3 days after reperfusion;	[139]
AMI ^6^	permanent ligation of LAD ^7^ coronary artery in Wistar rats	MSCs from human bone marrow	Four intramyocardial injections of 80 µg of EVs (2 × 10^6^ cell equivalents) from hypoxic- and serum-deprivated MSCs, 30 min after ligation	reduction of the infarct size, 28 days after AMI; improved cardiac function 2 and 4 weeks after AMI; promotion of blood vessel formation, 4 weeks after AMI;	[140]
AMI	permanent ligation of LAD coronary artery in Sprague-Dawley rats	MSCs from human umbilical cord	IV injection of 400 µg of exosomes from Akt-transfected MSCs, immediately after ligation	improved cardiac function, 1 and 5 weeks after Akt exosome treatment in AMI rats; increased blood vessel formation; key role of PDGF-D ^8^ in Akt exosomes-mediated angiogenesis;	[141]
AMI	permanent ligation of LAD coronary artery in C57/B6 mice	MSCs from mice heart	intramyocardial injection of 20 µL of EVs (1 × 10^12^ particles/mL), 10 min after ligation	improved cardiac function and attenuated cardiac fibrosis after C-MSCs^N1ICD 9^ transplantation in infarcted mouse hearts; decreased apoptosis and increased proliferation of cardiomyocytes, 24 h post-infarction; increased vessel density in peri-infarct area after 1 month;	[142]
AMI	permanent ligation of LAD coronary artery in Sprague-Dawley rats	MSCs from rat bone marrow	intramyocardial injection of 80 µg of exosomes, 60 min after ligation	improved cardiac function and reduced cardiac fibrosis, 4 weeks after AMI; increased blood vessel density; reduced infiltration of inflammatory cells;	[144]
AMI	permanent ligation of LAD coronary artery in C57BL/6J mice	MSCs from mouse bone marrow	intramyocardial injection of 1 µg of exosomes from ischemic pre-conditioned MSCs, immediately after ligation	reduced infarct size and cardiac fibrosis, 4 weeks after AMI; reduced apoptosis in the ischemic cardiomyocytes; the anti-apoptotic effect was mediated by miR-22 shuttled by MSC exosomes and targeting Mecp2 ^10^;	[145]
AMI	permanent ligation of LAD coronary artery in Sprague-Dawley rats	MSCs from human umbilical cord	IV injection of 400 µg of exosomes, immediately after ligation	improved cardiac function and reduced cardiac fibrosis, 4 weeks after exosomes infusion; reduced cardiomyocyte apoptosis in the infarcted myocardium; the anti-apoptotic Bcl-2 ^11^ was up-regulated following exosome injection;	[146]
AMI	permanent ligation of LAD coronary artery in Sprague-Dawley rats	MSCs from rat bone marrow	intramyocardial injection of 50 µL of exosomes from GATA-4 over-expressing MSCs (4 × 10^6^ cells), immediately after ligation	restoration of cardiac function and reduced infarct size, 4 weeks after AMI induction; reduced cardiomyocyte apoptosis; the anti-apoptotic effect was mediated by miR-19a shuttled by MSC exosomes and targeting PTEN ^12^ and BIM pro-apoptotic genes;	[147]
AMI	permanent ligation of LAD coronary artery in Sprague-Dawley rats	MSCs from rat bone marrow	intramyocardial injection of 20 µg (in 20 µL of PBS ^13^) of exosomes, immediately after ligation	restoration of cardiac function and reduced cardiac fibrosis, 7 days after AMI induction; reduced inflammation, 7 days after induced infarction; effects mediated by several miRNAs, some differentially expressed by MSC-derived exosomes and their parent cells;	[148]
I/R injury	30 min ischemia through ligation of LCA in Sprague-Dawley rats followed by 2 h reperfusion	MSCs from rat bone marrow	intramyocardial injection of 5 µg (in 10 µL of PBS) of exosomes, 5 min before reperfusion	decreased cardiomyocyte apoptosis, reduced infarct size, and improved cardiac function; increased expression of the autophagic protein LC3B ^14^; effects partly mediated by AMPK/mTOR and Akt/mTOR signaling.	[151]

^1^ ischemia/reperfusion; ^2^ left coronary artery; ^3^ intravenous; ^4^ glucogen synthase kinase-3; ^5^ c-Jun N-terminal kinase; ^6^ acute myocardial infarction; ^7^ left anterior descending; ^8^ platelet-derived growth factor D; ^9^ cardiac MSCs over-expressing Notch1 intracellular domain; ^10^ methyl cytosine-phosphate-guanine binding protein 2; ^11^ B cell lymphoma 2; ^12^ phosphatase and tensin homolog; ^13^ phosphate-buffered saline; ^14^ microtubule-associated protein 1 light chain 3 beta.
